# Utility of Artificial Intelligence in Antibiotic Development: Accelerating Discovery in the Age of Resistance

**DOI:** 10.7759/cureus.78296

**Published:** 2025-01-31

**Authors:** Esteban Zavaleta-Monestel, Carolina Rojas-Chinchilla, Jeimy Campos-Hernández, Ernesto Martínez-Vargas

**Affiliations:** 1 Research, Hospital Clinica Biblica, San Jose, CRI; 2 Pharmacy, Hospital Clínica Bíblica, San José, CRI

**Keywords:** antibiotic, antimicrobial resistance, artificial intelligence, drug discovery and development, multidrug-resistant bacteria

## Abstract

Antimicrobial resistance (AMR) is a growing public health issue, complicating the treatment of bacterial infections and increasing morbidity and mortality globally. This phenomenon, which occurs as a result of the ability of bacteria to adapt and evade conventional treatments, requires innovative strategies to address it. Artificial intelligence (AI) emerges as a transformative tool in this context, helping accelerate the identification of molecules with antimicrobial potential and optimize the design of new drugs. This article analyzes the usefulness of AI in antibiotic development, highlighting its benefits in terms of time, cost, and efficiency in the fight against resistant bacteria, as well as the challenges associated with its implementation in the biomedical field.

## Introduction and background

The term "antibiotic" has changed in meaning over time. Originally, it was limited to natural substances produced by microorganisms with antimicrobial properties. However, today, its definition has been considerably expanded to include any compound, whether natural or synthetic, capable of inhibiting or destroying bacteria. This shift reflects the increasing complexity in the development of strategies against bacterial infections, a problem that has acquired greater relevance in the context of antimicrobial resistance (AMR) [1,2].

In the early 20th century, before the discovery of penicillin in 1928, respiratory and gastrointestinal infections were responsible for one-third of deaths worldwide [3]. The incorporation of antibiotics into the clinical arsenal allowed a significant reduction in morbidity and mortality caused by bacterial infections, saving millions of lives. This positive impact laid the groundwork for the so-called 'Golden Age of Antibiotics,' a period between 1930 and 1960 when numerous compounds with a broad spectrum of action were identified [2].

During this period, advances made it possible to treat and cure infectious diseases such as pneumonia, tuberculosis, and post-surgical infections, among others. However, the emergence of antibiotic-resistant bacteria has drastically altered this scenario. Multidrug-resistant (MDR) bacteria are actively undermining the achievements of modern medicine and pose a significant threat to global health, with AMR now recognized as one of the greatest challenges of the 21st century [4-6]. Recent studies estimate that by 2050, resistant infections could be responsible for up to 10 million deaths annually, surpassing even deaths attributable to cancer. This alarming scenario underscores an unprecedented urgency to develop innovative strategies to address this crisis [7].

Artificial intelligence (AI) emerges as a transformative tool in this fight, with the ability to analyze large volumes of data and predict and model complex dynamics of AMR. Advances in machine and deep learning algorithms make it possible to identify new therapeutic targets, predict resistance patterns, and optimize the use of antimicrobials, opening new perspectives to face this global crisis. Previous studies have highlighted the potential of AI in drug discovery, focusing on its applications in accelerating target identification, reducing development timelines, and addressing the challenges of AMR [8-10]. However, there remains a need to synthesize recent advancements and analyze their practical implications in the context of combating MDR bacteria, justifying the relevance of this review in consolidating these developments [11]. 

## Review

Methodology

This article is a narrative review focused on assessing the role of AI in antibiotic development, particularly its impact on reducing time and cost, optimizing drug discovery processes, and addressing AMR. A structured methodology was followed to ensure the completeness and reproducibility of the results.

Search strategy

A systematic search was conducted in recognized electronic databases, including PubMed and Google Scholar, using a combination of search terms: “artificial intelligence,” “antibiotics,” “antimicrobial resistance,” “drug discovery,” “machine learning,” and “Halicin.” Boolean operators (AND, OR, and NOT) were used to refine the search. Filters were applied to select publications in English and Spanish, published between 2015 and 2025, encompassing a period marked by significant advancements in the application of AI to pharmaceutical research, prioritizing studies with recent data and high relevance to the topic.

Inclusion and exclusion criteria

We included studies exploring the application of AI in the discovery and development of antibiotics, focusing on its role in optimizing drug design, predicting resistance patterns, and accelerating antimicrobial innovation. Systematic reviews, case studies (e.g., Halicin), and experimental research with clear methodologies were considered. Exclusion criteria included non-peer-reviewed articles, duplicate studies, general AI research not related to antibiotics, and publications outside the defined time range.

Method of analysis

Data extracted from the selected studies were categorized based on thematic relevance, dividing them into sections that include the role of AI in drug discovery, case studies, efficiency improvements in development processes, and the challenges and limitations of AI implementation. The references were verified to ensure validity and represent diverse regional and global perspectives.

Selection Process

**Figure 1:**
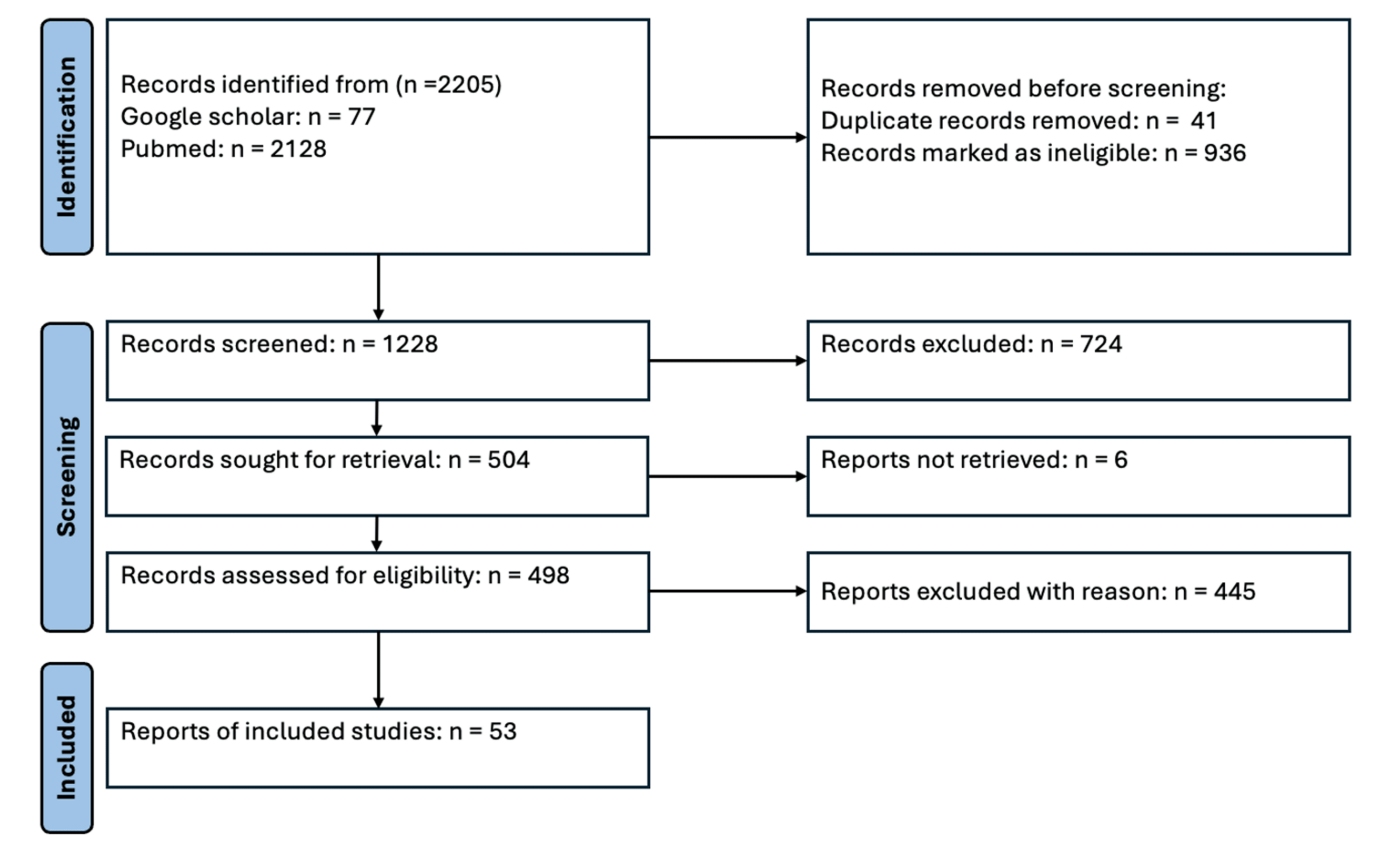
Flowchart of search results and selection of relevant articles

Initially, 2205 publications were identified. After removing duplicates and conducting a review of titles and abstracts, 498 articles were selected for full review. Ultimately, 53 studies met the inclusion criteria and formed the basis for the narrative synthesis. A flowchart detailing the selection and screening process is presented (Figure 1).

Antimicrobial resistance

AMR refers to the potential for microorganisms to thrive and continue to grow in the presence of drugs that are designed to kill them [12]. The problem stems from the selective pressure that antibiotics generate on bacterial populations. This process occurs when susceptible bacteria are killed by treatment, while bacteria with resistant mutations survive and reproduce, passing on their resistance genes to future generations. A notable example of this is the production of extended-spectrum beta-lactamases (ESBLs) by *Escherichia coli *and *Klebsiella pneumoniae*, which makes them resistant to antibiotics such as penicillins and second-, third- and fourth-generation cephalosporins and in some cases aztreonam [13,14].

The scientific community began to observe with growing concern the emergence of antibiotic-resistant bacteria, a phenomenon driven by the excessive and inappropriate use of these drugs, both in the medical field and in industry. This problem has become one of the main factors that favor the spread of resistant strains, constituting a serious threat to public health at a global level as a silent pandemic [12,15,16].

Methicillin-resistant Staphylococcus aureus (MRSA) is a clear example of the impact of bacterial resistance, having emerged in recent years as a significant cause of both hospital and community infections, with mortality rates ranging widely from 10% to 30% [17].

The resistance of Staphylococcus aureus to methicillin has dramatically transformed the way these infections are managed, affecting their sensitivity to antibiotics and treatment approaches. This change is due to the acquisition of the mecA gene, which encodes a protein called altered penicillin-binding protein (PBP2a). This protein has a low affinity for beta-lactam antibiotics, making these drugs ineffective [18].

The mecA gene is located on a mobile segment of DNA known as the Staphylococcal Cassette Chromosome mec (SCCmec), which is integrated into the bacterial chromosome. This genetic element can be transferred between bacteria with the help of recombinase enzymes. These enzymes facilitate their movement and integration into the chromosome of a recipient bacterium. Likewise, the resistance system replicates along with the bacterial genome during cell division, ensuring that the daughter bacteria inherit resistance [19,20].

**Figure 2:**
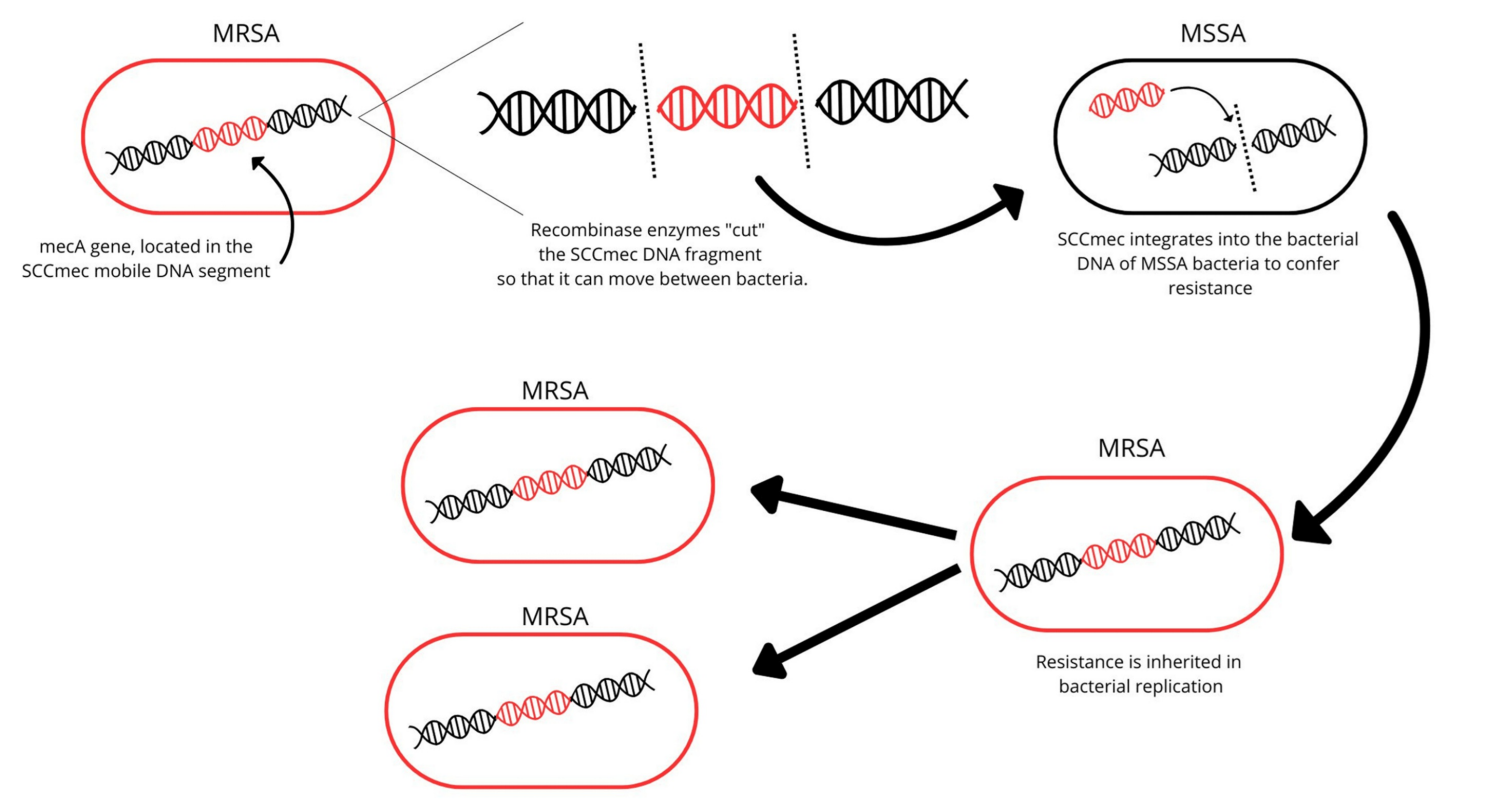
Transfer mechanism and integration of SCCmec into Staphylococcus aureus Created by the author. SCCmec: staphylococcal cassette hcromosome mec

This process of resistance propagation is illustrated schematically in the figure (Figure 2), which details the key steps of mecA gene transfer and inheritance.

Over time, these resistant bacteria become increasingly prevalent, making it difficult or even impossible to treat the infections they cause [6].

In 2019, antimicrobial-resistant infections were estimated to have caused approximately 1.27 million deaths globally. Of this total, 73% of deaths (927000 deaths) were attributed to a group of six main bacteria:* E. coli, Staphylococcus aureus, K. pneumoniae, Streptococcus pneumoniae, Acinetobacter baumannii and Pseudomonas aeruginosa* [21]. It is estimated that around 330000 of these deaths occurred in hospital settings because these bacteria possess a wide variety of resistance mechanisms that allow them to adapt, survive, and thrive in healthcare settings [22].

However, it is important to consider that these data may not fully reflect reality, due to the scarcity of information from low-income countries and may be an underestimate of the true burden of AMR. In these regions, the lack of a clear distinction between nosocomial and community-acquired infections, as well as the absence of standardized protocols for data collection and reporting, limits the accuracy and scope of global estimates of AMR [22].

The evolution of resistant bacteria is not a random process but the result of complex interactions between biological, ecological, and social factors. In this sense, understanding how these resistance mechanisms arise and spread is essential for designing more effective strategies to counteract them. Representing these interactions using visual schematics could be a useful tool to improve understanding of this complex problem [22].

Mechanisms of antimicrobial resistance

The mechanisms underlying antibiotic resistance present significant challenges to global health, as they enable bacteria to evade the effects of antimicrobial agents and persist despite treatment efforts. These mechanisms include enzymatic inactivation, structural modifications of therapeutic targets, alterations in permeability, overexpression of efflux pumps, and biofilm-mediated protection. Understanding the most clinically relevant resistance pathways and their prevalence in key pathogens is critical to developing effective strategies against MDR organisms [23]. As presented in Table 1, these mechanisms are detailed, including their biological basis, representative bacterial examples, and relevant epidemiological data, offering a comprehensive overview of their impact on AMR.

**Table 1:** Main mechanisms of bacterial resistance, clinical examples, and prevalence data ESBL: extended-spectrum beta-lactamase; PBP: penicillin-binding proteins; MRSA: methicillin-resistant Staphylococcus aureus; MDR: multidrug-resistant

Resistance mechanism	Description	Common example	Statistic data
ESBL production	Certain bacteria synthesize enzymes capable of hydrolyzing beta-lactam antibiotics, including penicillins and cephalosporins, rendering them ineffective	*E. coli *and *K. pneumoniae* harbor ESBL-producing genes such as CTX-M, SHV, or TEM	The prevalence of ESBLs producing *E. coli *in Latin America ranges from 1.7% to 16%. In the USA the prevalence is between 3% and 8% [13,24]
Alteration of PBPs	Structural modification of PBPs results in reduced affinity for beta-lactams, leading to treatment failure	MRSA harboring PBP2a encoded by the mecA gene	The prevalence of MRSA is estimated to be 10%-25% in Canada, 25%-50% in the US and Mexico, and exceeds 50% in countries such as Peru, Chile, Venezuela, and Brazil [25]
Efflux pumps	Overexpression of efflux systems actively transports antibiotics out of the bacterial cell, decreasing intracellular concentrations below therapeutic levels	*P. aeruginosa* with efflux pumps such as MexB, MexC, MexX, and MexY contributing to resistance to anti-pseudomonas antibiotics	MexY (74.6%) and MexB (69%) are the main efflux pumps in *P. aeruginosa*, associated with high resistance, particularly to ticarcillin (80%) [26]
Porin loss or modification	Alterations in porin expression reduce antibiotic uptake, limiting drug access to intracellular targets	*K. pneumoniae* resistant to carbapenems due to loss or mutation of porins such as OmpK35 and OmpK36	The overall prevalence among patients with *K. pneumoniae* is 26.69% [27]
Biofilm-mediated resistance	Biofilm formation provides a protective extracellular matrix that impairs antibiotic penetration and facilitates bacterial persistence	*P. aeruginosa* forming biofilms in chronic respiratory infections, such as in cystic fibrosis	Bacterial biofilms are responsible for nearly 80% of human infections [28]
Genetic mutations conferring resistance	Point mutations in bacterial genes alter target proteins or enhance enzymatic degradation of antibiotics, compromising their efficacy	*Mycobacterium tuberculosis *with mutations in the katG gene conferring high-level isoniazid resistance	Approximately 3.4% of new tuberculosis cases and 20% of previously treated cases are caused by *M. tuberculosis* strains MDR [29].

**Figure 3:**
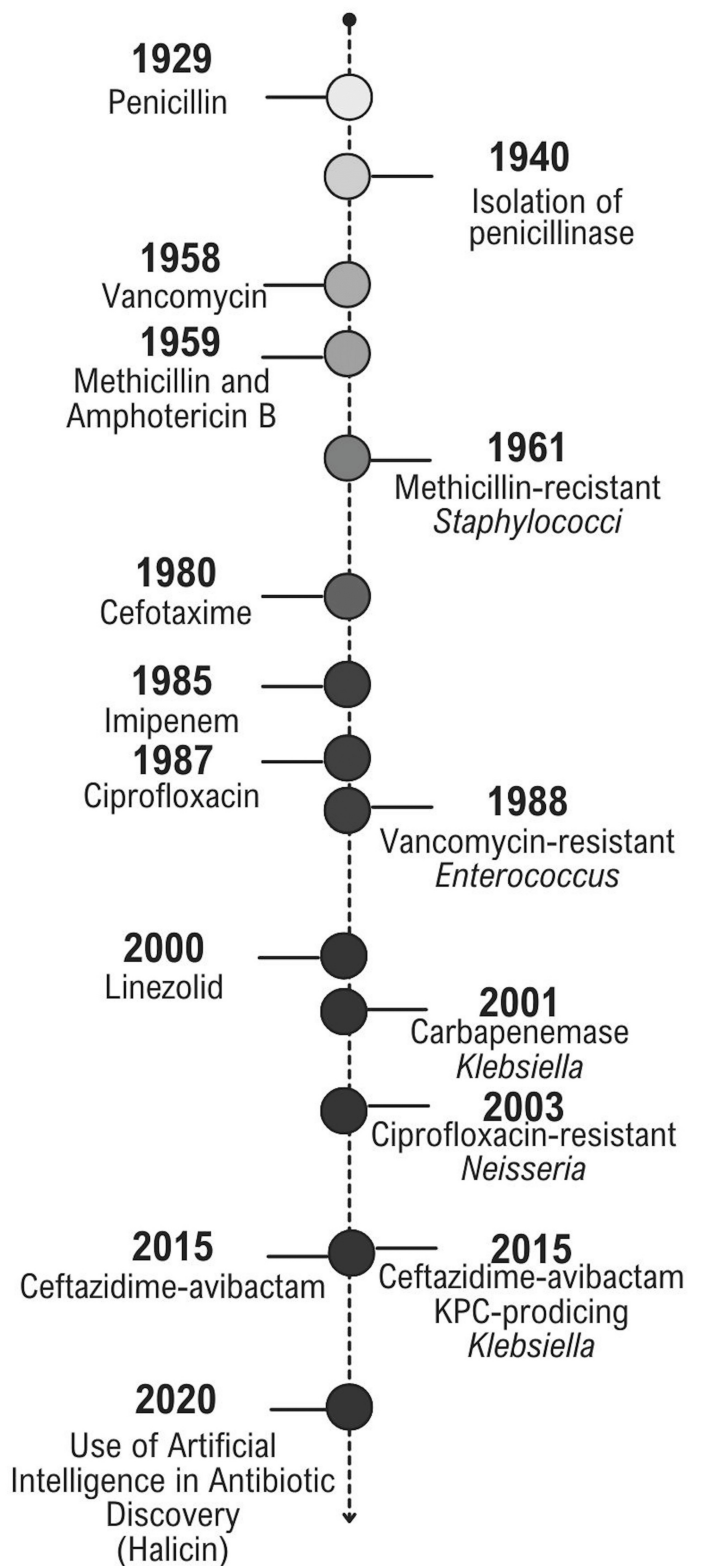
Temporal relationship between the introduction of antibiotics and the emergence Created by the author.

Figure 3 shows the development of antibiotics over the years versus the identification of resistance of bacteria to these antibiotics [30].

Rise of artificial intelligence

AI is emerging as a fundamental tool to combat the growing threat of bacteria resistant to current treatments. Its potential lies in its ability to process and analyze large amounts of data, identify complex patterns, and make accurate predictions [31].

Conventional methods for drug discovery with pharmacological potential are slow, expensive, and require many human resources and specialized equipment. In addition, the process is inefficient and has a high failure rate. It is estimated that between 2000 and 2015, 86% of drug candidates failed to meet the planned targets [32].

This generates a stagnation in the research and development of new antibiotics that has occurred since the 1980s, unlike other therapeutic areas, such as antihypertensives or cholesterol-lowering drugs, antibacterials present an investment landscape with high financial risk that not all developing industries want to take [3,33]. Computer-assisted automated techniques offer a faster and cheaper alternative, accelerating progress toward the preclinical and clinical phases [31].

AI in drug discovery makes it possible to exploit mathematical models that facilitate the processing of large amounts of data, predict molecular interactions, evaluate the efficiency of compounds, or generate new compounds with novel properties [34]. To do this, it is essential to train a deep learning algorithm that allows the identification of molecules with potential antimicrobial activity. This process involves feeding the program with detailed information about the molecular characteristics of a wide range of drugs and natural compounds, as well as data on their effectiveness in inhibiting bacterial growth. Once the training is complete, the algorithm can be applied to a library of compounds under investigation, evaluating their potential use in the treatment of various human diseases [35].

Based on these mathematical models analyzed through AI, in 2020 researchers at the Massachusetts Institute of Technology (MIT) identified an N-terminal kinase inhibitor c-Jun, renamed Halicin. It was initially being studied to treat diabetes, but it highlighted its broad-spectrum antimicrobial activity, potent activity against resistant strains of *E. coli*, as well as carbapenem-resistant Enterobacteriaceae, becoming the first antibacterial agent discovered through AI [36].

One of the main advantages of using AI in medicinal chemistry is its ability to predict both the efficacy and toxicity of potential chemical compounds. Traditional methods, on the other hand, often require extensive and laborious stages of experimentation, which involve a significant consumption of time and resources to determine the potential impact of the compounds on the human body [36].

**Figure 4:**
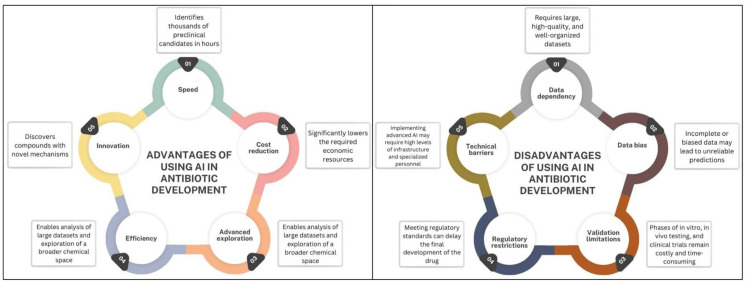
Main advantages and disadvantages of using AI in antibiotic development Created by the author. AI: artificial intelligence

Figure 4 presents a detailed summary of the advantages and disadvantages associated with the use of AI in drug development. This analysis highlights how these technologies not only optimize processes but also face limitations related to data quality and model interpretability.

A key aspect of the AI-driven revolution in antibiotic discovery lies in the dramatic reduction in the time it takes to bring a new drug to market. The traditional approach could take up to 12 years to discover an antibiotic and an additional three to six years to find a viable clinical candidate, and each drug requires an estimated $1 million to $2 billion during its development process [37]. This candidate then has to go through clinical trial phases (I, II, III) before approval. On the contrary, AI has shown extraordinary potential to accelerate this process. Thanks to its ability to process and analyze large amounts of data, AI can identify thousands or hundreds of thousands of preclinical candidates in a matter of hours, which previously required years of research. The integration of these technologies facilitates the exploration of a wider chemical space and the prediction of key molecular properties, significantly reducing the time and costs associated with the development of new antibiotics [38-40].

In the case of halicin, the identification process was completed in a few days to weeks, thanks to the ability of algorithms used in machine learning to quickly analyze large databases of chemical compounds. However, the full development of a drug, including preclinical and clinical testing, will still take a little longer depending on regulatory requirements and success in subsequent development. AI is especially valuable in the discovery and initial selection phases, where it is able to significantly accelerate this critical step [34,41].

Although it seems that everything around the use of AI to enhance the development of new molecules with pharmacological properties including antimicrobial activities, this technology still has limitations. AI models require a large, high-quality, and well-organized dataset to be properly trained. Incomplete or biased data generate results that are unreliable [42].

Likewise, as mentioned above, AI can quickly identify promising chemical compounds;89-0plokijuhygvcfx however, validation, which includes in vitro, in vivo, and clinical trials, remains a bottleneck in terms of time and costs for developers [43].

Halicin: the first antimicrobial discovered using artificial intelligence

Halicin (initially known as SU3327) is a molecule that was initially proposed as an alternative treatment for diabetes due to its ability to lower blood glucose levels as well as restore insulin sensitivity in mouse models suffering from type 2 diabetes, but it was disused before its arrival at the clinic. However, in 2020, through the application of AI and the use of machine learning algorithms, the ZINC15 database, which is a collection of almost 1500 million chemical compounds, was used to discover how halicin exhibited unique antibacterial activity against several strains of harmful bacteria, including MDR bacteria [44,45].

The mechanism of action of this drug differs from that of other antimicrobials. This molecule inhibits the transport of protons across the bacterial cell membrane. It inhibits the enzyme c-Jun N-terminal kinase (JNK), which is responsible for regulating other important cellular activities, including cell proliferation, differentiation, iron homeostasis, and apoptosis [44]. Inhibition of proton transport by halicin interferes with the ability of the bacterium to maintain a proton gradient necessary for ATP synthesis, stopping bacterial growth [34,45-47].

**Figure 5:**
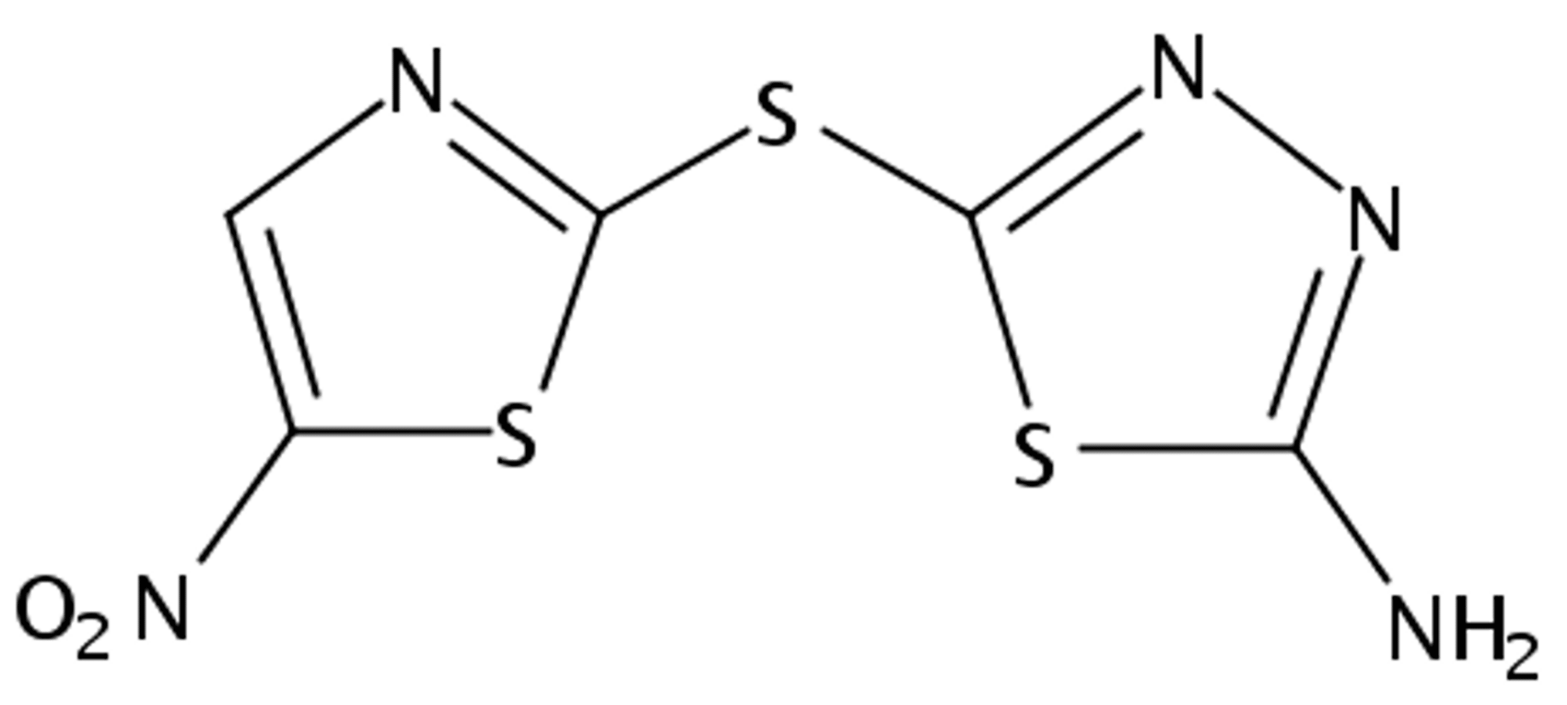
Chemical structure of halicin Created by the author with reference to the PubChem repository [48].

In 2022, studies were conducted proposing halicin as a possible treatment against SARS-CoV-2, due to the presence of its nitrothiazole group (Figure 5) and its ability to covalently bind to protease MPro, a key pharmacological target of the virus because of its essential role in the replication and pathogenesis of SARS-CoV-2. The results demonstrated that halicin had a strong inhibitory effect on MPro [44].

Recently, at the beginning of 2024, more studies have been carried out on the effectiveness of halicin against strains of Staphylococcus aureus methicillin-resistant (MRSA) using infected nematodes. The results indicate that halicin significantly prolongs the survival of infected nematodes, suggesting that it has potential in the treatment of MRSA infections. Still, physiological differences with humans can affect the extrapolation of results [49].

In laboratory tests, halicin has shown activity against gram-positive and gram-negative bacteria, including *E. coli, S. aureus,* and *A. baumannii*, even against strains of* A. baumannii* MDR, although in the latter case, higher concentrations are required. Therefore, it is essential to conduct extensive research to evaluate its potential clinical use in the treatment of MDR bacterial infections [44].

Potential clinical impact of using artificial intelligence

The development of antimicrobials using effective AI support could have significant repercussions in various areas. From a clinical point of view, it offers new therapeutic options to treat infections caused by MDR bacteria, reducing mortality and serious complications, especially in critical or immunocompromised patients. In addition, it could be integrated into combination treatments, optimizing current regimens to combat resistant infections [35,50,51].

In the field of clinical medicine, it would mitigate the global burden of resistant infectious diseases, identified by the WHO as one of the greatest threats to humanity due to the acceleration of the processes that these technologies promote. Its ability to prevent outbreaks and reduce the transmission of MDR bacteria would be a key advance in epidemic management. In addition, if its accessibility is guaranteed, it could benefit populations in countries with a high prevalence of resistant infections [52].

Finally, this breakthrough reinforces the transformative role of AI in drug discovery, establishing new paradigms in pharmaceutical design and popularizing drug repurposing. The success of halicin promises not only urgent clinical solutions but also reinvigorates antimicrobial research, marking a turning point in the fight against bacterial resistance [53].

## Conclusions

AI demonstrates significant potential as a transformative tool in the fight against AMR by enabling the rapid identification of therapeutic compounds, predicting key molecular properties, and reducing the time and cost associated with drug discovery and development. This review highlights how AI accelerates the exploration of novel antibiotic candidates, exemplified by breakthroughs such as halicin, which showcases its ability to discover innovative antimicrobial agents. Furthermore, AI enhances the efficiency of antimicrobial development by optimizing drug design, predicting resistance patterns, and enabling the repurposing of existing compounds. Despite its immense promise, the implementation of AI in antibiotic development faces challenges, such as the need for high-quality, comprehensive datasets, and the validation of AI-generated findings through rigorous experimental trials. Addressing these hurdles is crucial to harnessing AI’s full potential as a driving force in pharmaceutical innovation, ultimately transforming clinical practices, reducing the spread of resistant infections, and offering powerful strategies to combat one of the most pressing global health threats of our time.
